# Absence of toxicity with hypofractionated 3-dimensional radiation therapy for inoperable, early stage non-small cell lung cancer

**DOI:** 10.1186/1748-717X-1-42

**Published:** 2006-11-01

**Authors:** Sergio L Faria, Luis Souhami, Lorraine Portelance, Marie Duclos, Te Vuong, David Small, Carolyn R Freeman

**Affiliations:** 1 Department of Radiation Oncology, McGill University Health Centre, Montreal, Canada; 2 Pulmonary Division of the Jewish General Hospital, Montreal, Canada

## Abstract

**Purpose:**

Hypofractionated radiotherapy may overcome repopulation in rapidly proliferating tumors such as lung cancer. It is more convenient for the patients and reduces health care costs. This study reports our results on patients with medically inoperable, early stage, non-small cell lung cancer (NSCLC) treated with hypofractionation.

**Materials and methods:**

Stage T1-2N0 NSCLC patients were treated with hypofractionation alone, 52.5 Gy/15 fractions, in 3 weeks, with 3-dimensional conformal planning. T1-2N1 patients with the hilar lymphnode close to the primary tumor were also eligible for this treatment. We did not use any approach to reduce respiratory motion, but it was monitored in all patients. Elective nodal radiotherapy was not performed. Routine follow up included assessment for acute and late toxicity and radiological tumor response. Median follow up time was 29 months for the surviving patients.

**Results:**

Thirty-two patients with a median age of 76 years, T1 = 15 and T2 = 17, were treated. Median planning target volume (PTV) volume was 150cc and median V16 of both lungs was 13%. The most important finding of this study is that toxicity was minimal. Two patients had grade ≤ 2 acute pneumonitis and 3 had mild (grade 1) acute esophagitis. There was no late toxicity. Actuarial 1 and 2-year overall survival rates are 78% and 56%, cancer specific survival rates (CSS) are 90% and 74%, and local relapse free survival rates are 93% and 76% respectively.

**Conclusion:**

3-D planning, involved field hypofractionation at a dose of 52.5 Gy in 15 daily fractions is safe, well tolerated and easy radiation treatment for medically inoperable lung cancer patients. It shortens by half the traditional treatment. Results compare favorably with previously published studies. Further studies are needed to compare similar technique with other treatments such as surgery and stereotactic radiotherapy.

## Background

The use of accelerated hypofractionated radiation therapy is an attractive option for patients with early stage non-small cell lung cancer (NSCLC) who are not surgical candidates. Although primarily appealing for patients with tumors that are rapidly proliferating such as NSCLC because of a possible biologic advantage [1-4], hypofractionation is also of interest because it requires half of the number of hospital visits, making it very convenient for sick/elderly patients, reduces health care costs and frees up resources for other patients

The optimal accelerated regimen of conformal 3-dimensional hypofractionated radiation therapy (3DHRT) for this group of patients is yet to be defined. Hypofractionation may result in an increase of normal tissue effects and a careful evaluation of acute and late toxicity is essential. The current challenge is to find the best balance between an optimal tumoricidal dose and an acceptable toxicity rate.

Since 2002 we have treated patients with inoperable early stage NSCLC with 3DHRT. This study reports the results of this experience.

## Materials and methods

All stage T1-2, N0 lung cancer patients, medically unfit for surgery, received this 3DHRT. T1-2, N1 patients were also allowed to receive the hypofractionated regimen if the hilar region was in close proximity to the primary tumor. There was no exclusion based on age, size of the tumor or respiratory function. They were staged with chest X-ray and CT-scan of the chest including the upper part of the abdomen. Eight patients only had also PET/CT scans pre-treatment. CT-scan of brain, abdominal imaging and bone scans were ordered only for patients with a suspicious sign or symptom of distant metastases.

**Figure 1:**
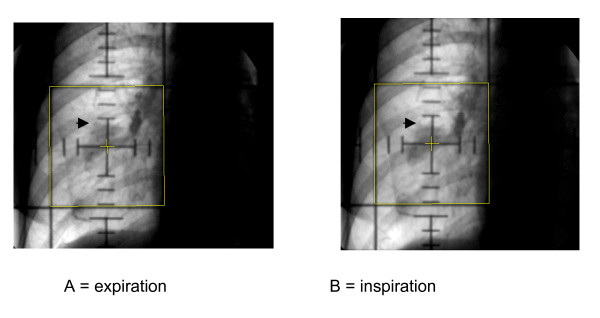
Example of a lung tumor in the right lower lobe easily seen by fluoroscopy with normal breathing during expiration (A) and inspiration (B). The radiation field, in spite of the motion, encompasses appropriately the tumor. The maximum displacement is only 4 mm.

They all received 3DHRT alone to a total dose of 52.5 Gy given in 3 weeks, in 15 daily fractions of 3.5 Gy prescribed at the isocenter, using 18 MV photons, without any device to reduce respiratory motion. Breathing motion was monitored in all patients by fluoroscopy (Figure 1) and/or by using multiple electronic cine-portals during treatment. Dose was prescribed to the isocenter without lung correction for inhomogeneity (it is the routine practice in our Department). Gross tumor volume (GTV) encompassed only the radiologically visible tumor as seen by the chest CT with the lung window. Planning target volume (PTV) was GTV plus a 10–15 mm margin in all directions. Elective nodal radiotherapy was not performed. The treatment planning ensured that the esophagus, heart and spinal cord received the minimum possible dose, but always less than 50% of the total prescribed tumor dose. Acute toxicity was prospectively assessed for lung, esophagus, and skin using the RTOG acute radiation morbidity scoring criteria every week during treatment. Late lung toxicity was evaluated with a modified scoring system considering only the lung symptoms, as summarized in Table 1. Radiographic abnormalities alone in asymptomatic patients were not considered to represent late lung toxicity. Late toxicity for other organs was evaluated using the Common Toxicity Criteria (CTC) version 2.

**Table 1:** Symptoms only scoring used for late lung toxicity (modified from the Common Toxicity Criteria (CTC) version 2 – Lung)

0	1	2	3	4	5
No increase in lung symptoms	Increase in lung symptoms due to RT but not requiring steroids	Same but steroids are required	Oxygen is needed	Assisted ventilation is required	Death related to radiation

Follow-up was done every 3–4 months with chest X-rays performed routinely at all visits. CT scans of the chest were performed in patients with abnormal x-ray findings, symptoms, or suspicion of disease progression. Patients were considered as dying from NSCLC if they had evidence of active cancer, either locally or distantly, before death.

First recurrence was classified as 1) *local *if it was at the site of the primary tumor, 2) *regional *if it was inside the chest but not local, or 3) *distant *when it occurred in any other site outside the chest. The date of first recurrence was that of the confirmation of recurrence (usually by imaging). Time zero was the date of the first radiotherapy day. Survival curves were constructed using the Kaplan-Meier method. This retrospective study was performed according to the guidelines of the McGill University Health Centre Ethical Committee.

## Results

Between October 2002 and June 2004, 32 patients entered in the 3DHRT program. None received chemotherapy. There were 23 (72%) males and 9 (28%) females. The median age at diagnosis was 76 years (range 56–90). Median FEV1 = 1.08 (range: 0.51–2.80). Fifteen cases (47%) were T1 and 17 (53%) were T2 because had more than 3 cm in greatest dimension. Twenty patients had confirmed histology of NSCLC. For the remaining 12 patients, the presumptive diagnosis of NSCLC was made by a multidisciplinary team based on the medical history and imaging findings. In these 12 cases biopsy was either inconclusive or was not tried because the procedure was felt to carry a high risk of complication in these patients.

The majority of patients (94%) were N0 by CT scan. Only two patients (6%) were considered to have N1 disease close to the primary lung tumor. In 19 cases (59%) the tumor was in the right lung. In 22 cases (69%) the tumor was located in the upper lobes.

**Figure 2:**
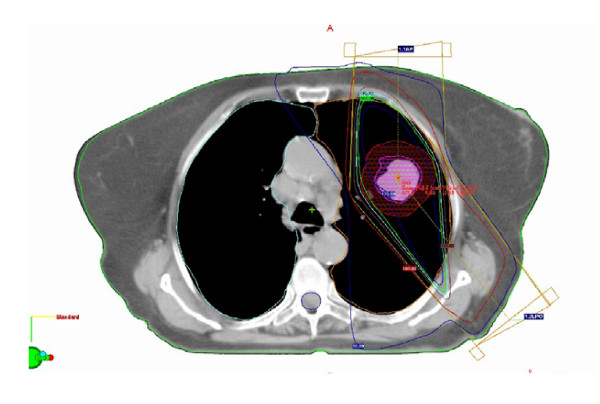
Example of typical 3-dimensional planning with 2 fields avoiding the esophagus, heart and spinal cord.

Radiotherapy was delivered mostly with either two fields (either opposed or wedge pair) (Figure 2) in 17 cases (53%) or 3 fields in 13 (41%) cases. Although less conformal, we preferred using 2 or 3 fields to avoid irradiating more normal lung and to facilitate monitoring the respiratory motion.

The median area of the fields was 72 cm^2 ^(typically 8 cm × 9 cm) and the median PTV volume was 150 cm^3 ^(range: 28–1110). The median V16 value for both lungs (PTV/GTV was not excluded from the V16 determination) was 13% (range: 3 – 29).

**Figure 3:**
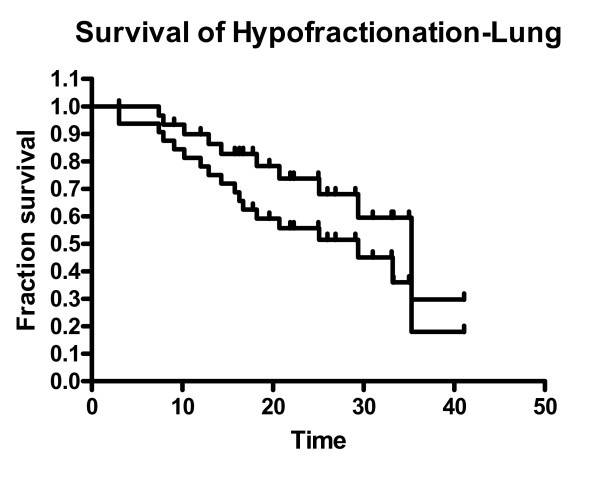
Overall (OS) (lower) and Cancer Specific (CSS) (upper) actuarial survival curves.

No patient was lost to follow up. As May 2006, the median follow-up time was of 29 months for the surviving patients and 21 months (range: 3 – 41 months) for the whole group. Eighteen patients had died, but only 10 due to lung cancer progression. Sixteen patients developed some form of recurrent disease. The first site of failure was as follows: 5 local only, 2 local + regional, 5 regional only, 1 regional + distant and 3 distant only. Actuarial 1 and 2-year overall survival rates are 78% and 56%, cancer specific survival rates (CSS) are 90% and 74% (Figure 3), and local relapse free survival rates are 93% and 76% respectively. Median overall survival and cancer specific survival are 29 and 35 months respectively.

Toxicity was mostly non-existent. One patient had grade 1 and another grade 2 acute pneumonitis and 3 patients had very mild (grade 1) acute esophagitis. There was no acute skin toxicity. Concerning late toxicity results were even better. No late toxicity has been observed in the esophagus, skin, subcutaneous tissue and even lungs (remembering that radiographic abnormalities alone were not considered as late lung toxicity).

## Discussion

According to the model suggested by Abratt and Hunter [5], assuming an effective doubling time of 3 days and that repopulation in NSCLC commences in 21 days, 52.5 Gy in 15 daily fractions of 3.5 Gy given in 3 weeks would be biologically equivalent to 78 Gy in 39 daily fractions of 2 Gy, given over 8 weeks. Table 2 compares some hypofractionated regimens according to that model.

**Table 2:** Comparison of different radiation therapy fractionations according to Abratt model.

	**Dose per fraction**	**No of fractions**	**Time (days)**	**Total Dose (Gy)**	**ID2 acute reaction**	**TED**	**ID2 late reaction**
McGill	3.5 Gy	15	19	52.5	59	59	68
Standard 2 Gy	2 Gy	39	53	78	78	59	78
Sunnybrook^13^	4 Gy	12	16	48	56	56	67
Wales^14^	2.75	20	26	55	58	55	60

ID2 = isoeffective dose at 2 Gy per fraction; acute reaction α/β = 10; late reaction α/β = 3. **TED **= tumor effect dose (used to indicate the ID2 modified for overall treatment time to account for repopulation) (5).

The contribution of the present study is mostly related to toxicity, which has always been a concern with any hypofractionated radiation treatment. In our present series acute toxicity was prospectively evaluated and found to be minimal with the dose of 52.5 Gy delivered in 3 weeks. To date there has been no late toxicity, showing that this regimen is a very safe radiation treatment. The fact that the patients did not have elective mediastinal nodal irradiation [6,7] and received essentially no irradiation to the esophagus, heart, and spinal cord, likely explains their very good tolerance to the treatment in spite of being patients with co-morbid diseases.

Co-morbid conditions have been shown to affect prognosis in early stage NSCLC [8]. Unfortunately, the typical early stage NSCLC patients referred for curative radiation therapy have grave impairment of pulmonary function, serious cardiovascular disease [9], are often imprecisely staged [10], and up to 43% of the patients die from inter-current diseases [11]. In our present cohort of patients the situation is not different. Some of our patients were so frail that procedures like a needle biopsy could not be performed.

In general, patients treated with radiation alone are older when compared to patients treated by surgery: the median age in our present group is 76 years compared to 65 years in surgical series [12]. This poor general physical condition explains the fact that 7 of our patients died within only 12 months after the radiation therapy (four of them with no evidence of tumor progression) making it difficult to evaluate long-term outcome in this group of patients.

Local control is the main purpose of localized radiation therapy, particularly for early stage NSCLC patients where local relapse is considered to be the most common failure pattern after radiation therapy alone [9,11]. We need to have in mind that lung is very sensitive to radiation and some times it is difficult to differentiate fibrosis from local relapse. This difficulty likely explains the wide range of local failure, reported from 6.4% to 70% [9]. In our present series 7/32 patients (22%) were considered to have local relapse (alone or with other metastases), suggesting that this regimen seems to give local control at least similar to what has been reported with standard fractionation [9-11].

Cheung et al [13] reported 1 and 2-year CSS of 89.8% and 54.1% respectively, with the use of hypofractionated radiation therapy alone in early stage NSCLC, dose of 48 Gy in 12 fractions, using 2D and 3DHRT in 33 patients.

The largest series of early stage NSCLC patients treated with accelerated hypofractionated radical radiotherapy using 3D planning comes from Wales [14]. Using 2-D and 3-D planning, 112 patients clinically staged as I/II, most of them receiving the dose of 50 Gy in 20 daily fractions, had an overall median survival of 23.5 months. The authors do not mention the rate of local control for this early stage group of patients; toxicity was evaluated retrospectively and no severe grade 3/4 toxicity was recorded.

We did not use any special device to either decrease inspiratory motion or to deliver radiation in fixed phases of the breathing cycle. However, we carefully monitored the tumor motion of our patients with fluoroscopy or electronic portal images to confirm that the GTV was inside the treatment fields (Figure 1). We cannot compare this relatively simple 3-D planning technique with stereotactic body radiation therapy. The latter is a promising alternative, but it requires significant technical advances to minimize organ motion due to respiration and in the use of tumor imaging to guide the administration of the radiation treatment [15-17]. It is still not available in most institutions and for this reason we believe there is plenty of space for 3DHRT.

## Conclusion

Three-dimensional involved field hypofrationated radiotherapy, at a dose of 52.5 Gy in 15 fractions of 3.5 Gy, given in 3 weeks, shortens by half the traditional treatment duration, is safe and very well tolerated by patients with medically inoperable early NSCLC. The technique does not require any special device to deliver the radiation treatment allowing any service of radiation oncology with 3-D planning to do it. Results of 2-year local relapse free survival of 76% and CSS of 74% respectively, compare favorably with other published results. Based on this present and on other Canadian experiences [13] the National Cancer Institute of Canada (NCIC) recently started a phase II trial (BR-25) using similar hypofractionated technique giving the dose of 60 Gy in 15 fractions for this group of NSCLC patients.

## Competing interests

The author(s) declare that they have no competing interests.
